# A novel system of bacterial cell division arrest implicated in horizontal transmission of an integrative and conjugative element

**DOI:** 10.1371/journal.pgen.1008445

**Published:** 2019-10-14

**Authors:** Sotaro Takano, Kohei Fukuda, Akiko Koto, Ryo Miyazaki

**Affiliations:** 1 Bioproduction Research Institute, National Institute of Advanced Industrial Science and Technology (AIST), Tsukuba, Japan; 2 Department of Bioscience, Graduate School of Science and Technology, Shizuoka University, Hamamatsu, Japan; 3 Computational Bio Big Data Open Innovation Laboratory (CBBD-OIL), AIST, Tokyo, Japan; 4 Faculty of Life and Environmental Sciences, University of Tsukuba, Tsukuba, Japan; Université Paris Descartes, INSERM U1001, FRANCE

## Abstract

Integrative and conjugative elements (ICEs) are widespread mobile DNA elements in the prokaryotic world. ICEs are usually retained within the bacterial chromosome, but can be excised and transferred from a donor to a new recipient cell, even of another species. Horizontal transmission of ICE*clc*, a prevalent ICE in proteobacteria, only occurs from developed specialized transfer competent (tc) cells in the donor population. tc cells become entirely dedicated to the ICE transmission at the cost of cell proliferation. The cell growth impairment is mediated by two ICE*clc* located genes, *parA* and *shi*, but the mechanistic and dynamic details of this process are unknown. To better understand the function of ParA and Shi, we followed their intracellular behavior from fluorescent protein fusions, and studied host cell division at single-cell level. Superresolution imaging revealed that ParA-mCherry colocalized with the host nucleoid while Shi-GFP was enriched at the membrane during the growth impairment. Despite being enriched at different cellular locations, the two proteins showed *in vivo* interactions, and mutations in the Walker A motif of ParA dislocalized both ParA and Shi. In addition, ParA mutations in the ATPase motif abolished the growth arrest on the host cell. Time-lapse microscopy revealed that ParA and Shi initially delay cell division, suggesting an extension of the S phase of cells, but eventually completely inhibit cell elongation. The *parA-shi* locus is highly conserved in other ICE*clc*-related elements, and expressing ParA-Shi from ICE*clc* in other proteobacterial species caused similar growth arrest, suggesting that the system functions similarly across hosts. The results of our study provide mechanistic insight into the novel and unique system on ICEs and help to understand such epistatic interaction between ICE genes and host physiology that entails efficient horizontal gene transfer.

## Author summary

Horizontal gene transfer is a major driving force for bacterial evolution, which is frequently mediated by mobile DNA vectors, such as plasmids and bacteriophages. Integrative and conjugative elements (ICEs) are relatively newly discovered mobile vectors, which are integrated in a host chromosome but under certain conditions can be excised and transferred from the host to a new recipient cell via conjugation. Recent genomic studies estimated that ICEs are widespread among bacteria, raising the question of the factors promoting their wide prevalence. One of the characteristics of ICE*clc*, an ICE model in proteobacteria, is that it develops specialized cells, which become entirely dedicated to the ICE horizontal transmission by repressing their proliferation. Here, we qualitatively and quantitatively describe functions of two key ICE proteins, which control cell growth arrest in transfer-dedicated cells. The two proteins are widely conserved among other ICEs of the same family, suggesting that the system of growth arrest in transfer-dedicated cells has been co-selected as a means for efficient ICE horizontal transmission.

## Introduction

Horizontal gene transfer is a pivotal event for prokaryotic evolution, since large pieces of DNA can be exchanged among bacterial species. Horizontal gene transfer is frequently, but not exclusively, mediated by mobile DNA vectors such as conjugative plasmids and bacteriophages. Integrative and conjugative elements (ICEs) form an additional class of ubiquitous mobile vectors that are usually integrated within the bacterial chromosome but under certain conditions can be excised and transferred from the donor to other bacterial cells where they again integrate into the new host chromosomes [1–3]. Only a few ICEs have been experimentally characterized from both gram-positive and gram-negative bacteria [4], but many more potential ICEs have been inferred from bioinformatic approaches [5–7]. Like other mobile vectors, ICEs often carry cargo genes that encode distinct adaptive features, such as antibiotic resistance and metabolic functions, which may give selective advantage to the ICE-bearing host [4]. It has been estimated that conjugative systems of ICEs are more abundant among bacteria than those of plasmids [6].

While the mechanistic basis for the pervasiveness of ICEs in the prokaryotic world is still poorly understood, part of it can be explained by their characteristic lifestyle. Once inserted in the host chromosome, ICEs are faithfully copied in every dividing cell. As long as the integrated ICE does not impose major fitness costs on the host, or even provides selective benefit by its cargo genes, it is stably maintained in the cells. Indeed, previous studies have reported very limited fitness costs (below 1%) and direct benefits from the ‘silenced’ integrated form of the ICE on the whole population [8,9]. In contrast, the process of horizontal transmission itself seems costly on fitness, because the ICE must excise from the chromosome and induce its donor cell to produce a DNA transfer machinery with associated factors [10,11]. The fact that horizontal transmission of ICE typically occurs at a very low rate (varied from 10^−2^ to 10^−7^ per donor cell, depending on ICEs) suggests that it is disadvantageous on fitness [1]. A key question in the ICE-host partnership is thus to understand how it has evolved to deal with fitness cost, while guaranteeing efficient ICE horizontal distribution.

**Fig 1:**
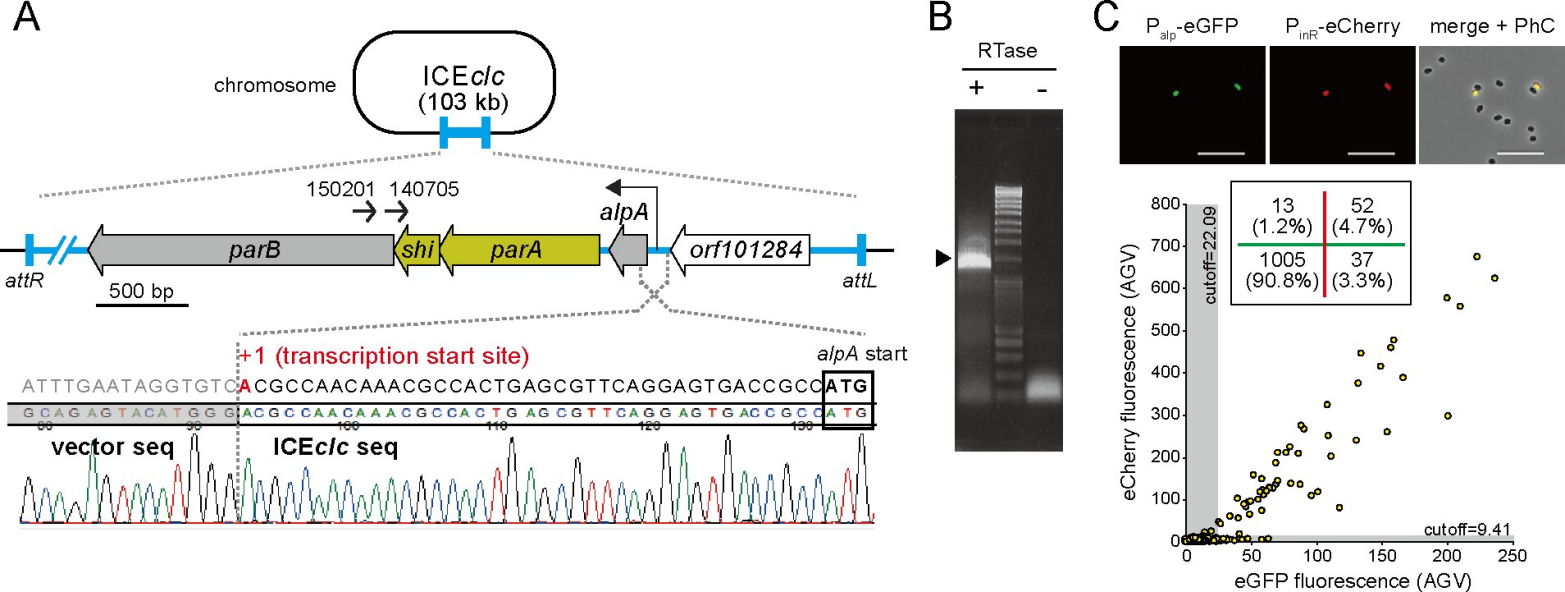
The *parA-shi* region of ICE*clc*. (A) Location of *parA-shi* gene cluster on ICE*clc* nearby the *attL* end. Positions and directions of primers used for 1st (150201) and 2nd (140705) PCRs to amplify 5’RACE products are indicated as small arrows. Lower part shows the sequence of a 1.4-kb 5’RACE product which determined the transcription starting site (adenine in red) of the *parA-shi* cluster. The boundary between the 5’RACE product and the cloning vector used is illustrated. (B) Agarose gel electrophoresis of the 2nd PCR product in 5’RACE. A specific 1.4-kb product is indicated by an arrow head. Presence or absence of the reverse transcriptase in each cDNA synthesis reaction is shown by ‘+’ or ‘-‘, respectively. (C) Representative micrographs and average fluorescence intensities of *P*. *putida* single cells carrying ICE*clc* and single insertions of P_alp_-*egfp* and P_inR_-*echerry*. Images show eGFP (green), eCherry (red), and merged with phase–contrast channels. Scale bar indicates 10 μm. Scatter plot showing correlation between single cell eCherry versus eGFP fluorescence values (circles) in stationary phase 3CBA-grown cultures. Grey zones indicate cells below cutoff values of the fluorescence expressions (for calculation, see S2 Fig). The inset in the scatter plot shows the number (and proportion) of cells separated by the cutoff values. Green and red lines indicate eGFP and eCherry cutoff values, respectively. Note that 4.7% of the 1,107 cells tested express both fluorescence proteins.

We study ICE*clc*, an 103-kb ICE originally found in *Pseudomonas knackmussii* B13 and widely distributed in proteobacteria [12,13], as an experimental model to understand evolution and adaptation of ICEs with host bacteria. We have shown that horizontal transmission of ICE*clc* necessitates the development of the host bacterial cells into a transfer competence (tc) state, which occur in only 3–5% of the stationary phase cells in a clonal population [14]. The tc cells arise as a consequence of stochastic intracellular variability of regulatory molecules and subsequent bistable expression of ICE*clc* genes in stationary phase [11,15,16]. Recent experimental data suggested that excision and transfer do not occur in stationary phase cells, but only when tc cells have been presented with new nutrients [17]. This suggested that ICE*clc* transfer is energetically costly for individual donor cells and thus restricted in a small subpopulation. Intriguingly, tc cells do not only commit to ICE*clc* transfer, but their proliferation is also impaired by simultaneous expression of two ICE*clc* genes, *parA* and *shi*, annotated as encoding a Walker ATPase and a hypothetical protein, respectively [14]. The *parA* and *shi* genes are located within a gene cluster adjacent to the *attL* end, one of the boundaries between the host chromosome and integrated ICE*clc* (Fig 1A). Expression of the two genes alone in a heterologous host without ICE is sufficient to induce cell growth arrest and abnormal cell morphologies, whereas their deletion in ICE*clc* abolishes the growth inhibition but, importantly, reduces the ICE transfer frequency [14]. Since the transfer frequency of ICE*clc* is relatively high (10^−2^ per all donor cells) among ICEs but the transfer actually occurs from 3–5% of the cells, the effect of *parA* and *shi* is crucial in the tc cells. The growth impairment by *parA* and *shi* is thus thought to benefit the overall transfer success of ICE*clc*; it is an adaptive system to increase its transfer frequency but not essential for transfer [14,17,18]. However, the mechanistic details of the inhibitory process and the dynamic action of the two proteins in the tc cell are unknown.

The goal of the underlying work was to study molecular characteristics of ParA and Shi, and their inhibitory effects on host cell growth. We showed that Shi is a protein, and developed fluorescence reporter strains to visualize intracellular behavior of ParA and Shi at the single-cell level. We made mutations in ParA inactivating its ATPase function and studied *in vivo* interactions between ParA and Shi by immuno-precipitation. We then tested whether growth inhibition by ParA/Shi may be the result of blocking of cell division or of cell elongation, by quantifying cell growth parameters in time-lapse microscopy. Time-lapse data favor the model that the two proteins inhibit cell division via blocking chromosome segregation, but also indicated that they halt cellular elongation in later stages of growth. The results of our study give insight into the mechanisms of a novel cellular growth inhibition system, which is highly conserved in ICE*clc*-related elements.

## Results

### Genetic characterization of *parA-shi* locus

While *parA* of ICE*clc* has been annotated as encoding a partitioning ATPase, *shi* is predicted to be a short open reading frame (258 bp) for a hypothetical protein overlapping 17 bp and 8 bp with up- and downstream genes, *parA* and *parB*, respectively (Fig 1A). We thus first examined whether the *shi* gene encodes a protein or acts as a non-coding RNA or a *cis*-acting element. Two different *shi* mutants of which translations were compromised either by the replacement of the start codon ATG with ACG (*shi* mt1) or by the substitution of leucine 7 to an opal mutation (*shi* mt2) were cloned on the pME6032 plasmid in *P*. *putida* together with *parA* under the control of LacI^q^/P_tac_ system. While the induction of wild-type *shi* with *parA* led to severe growth impairment as observed in the previous study [14], the two mutations caused no growth effects (S1 Fig). This revealed that *shi* encodes a protein acting together with ParA, and inhibits cell growth. To determine the transcriptional starting site of the *parA*-*shi* locus, we performed 5’ rapid amplification of cDNA ends (5’RACE) with reverse primers specifically annealing to the *parB* region. Subcloning and sequencing of a specific 1.4-kb 5’RACE product revealed a transcription start at an adenine 39 bases upstream of the *alpA* gene, encoding a putative transcriptional regulator (Fig 1A and 1B). This indicates that the gene cluster at least from *alpA* to *parB* are cotranscribed as an operon from the promoter region (hereafter, P_alp_) between *alpA* and *orf101284*.

We then measured the P_alp_ activity in tc cells of *P*. *putida* carrying ICE*clc*. The promoter was fused with *egfp* and subsequently integrated by mini-Tn*7* delivery into the chromosome, with another single copy insertion of an P_inR_-*echerry* fusion as a marker for tc cells [16]. Both eGFP and eCherry were coexpressed in ~5% of stationary phase cells, demonstrating that P_alp_ was indeed activated in tc cells (Fig 1C and S2A Fig). In addition, we found that ~3% of cells which did not yet express eCherry weakly expressed eGFP (Fig 1C). This suggests that, in the bistability network of ICE*clc*, P_alp_ is activated prior to P_inR_. One may assume a regulatory role of the ParA-Shi system on the tc cell development. We thus introduced a single copy of an P_inR_-*egfp* fusion [16] into *P*. *putida* carrying a *shi*-deleted ICE*clc* copy [14], to quantify the number of tc cells. The proportion of tc cells was indistinguishable from that with wild-type ICE*clc* (S2B Fig). This result shows that the ParA-Shi-mediated growth impairment does not affect the development of tc cells.

We confirmed that the expression level from P_alp_ in tc cells was comparable to that from the induced P_tac_ promoter on pME6032 (S2C Fig), indicating that the vector system can be used for further analysis in this study.

### *in vivo* localization and interaction of ParA and Shi proteins

**Fig 2:**
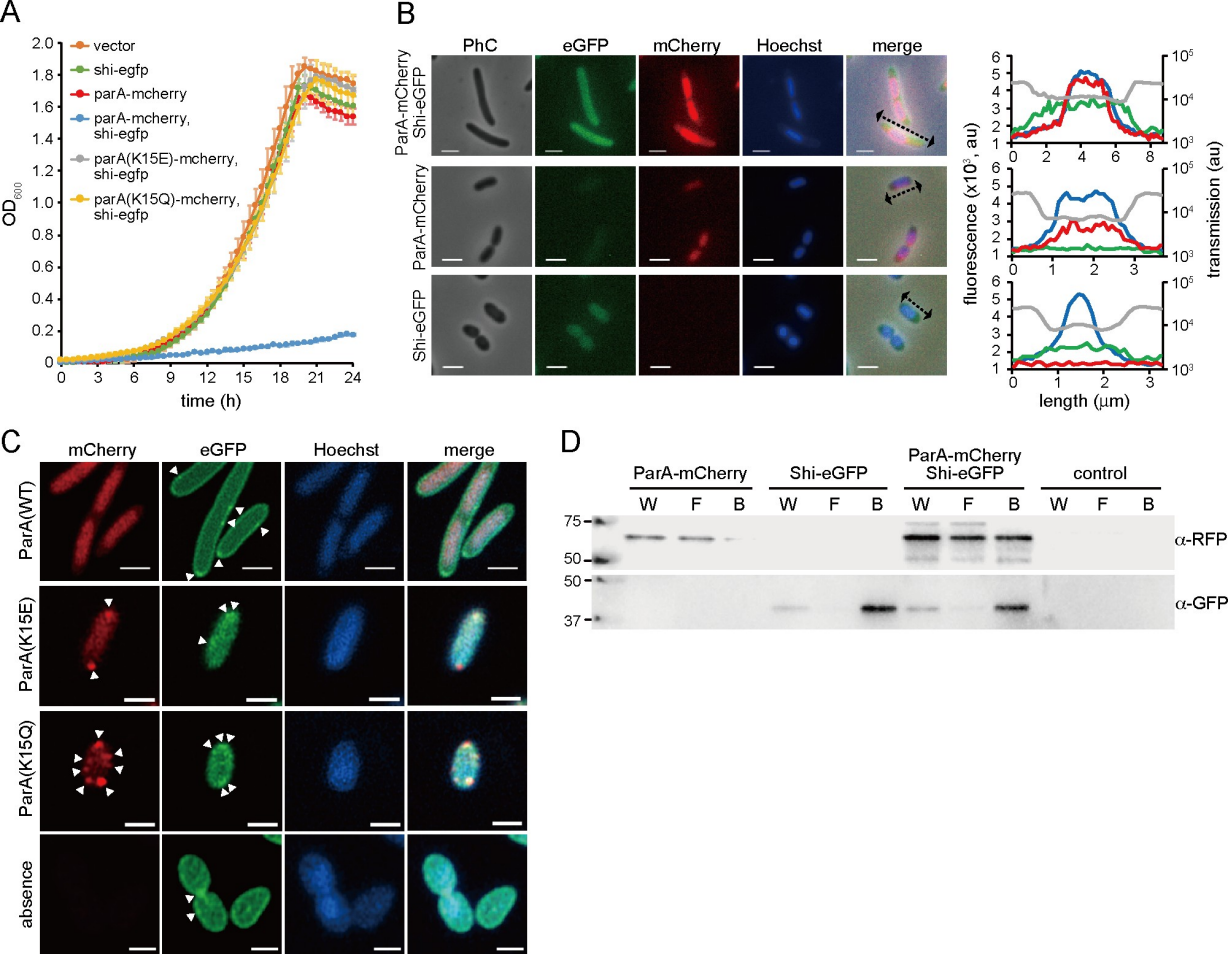
Subcellular localization and interaction of ParA and Shi proteins in *P*. *putida* without ICE*clc*. (A) Population growth of cells carrying pME6032 derivatives. Cells are cultured with IPTG, and their turbidity is measured. Genetic information expressed from P_tac_ promoter on the vector is indicated. Error bars represent standard deviation (SD) from the mean in triplicate assays. Vector, pME6032 (empty). (B) Representative micrographs and fluorescence profiles of single cells expressing either ParA-mCherry or Shi-eGFP, or both. Phase contrast (PhC) and fluorescence (eGFP, mCherry, and Hoechst33342) images are acquired at 4h after IPTG induction. Scale bar indicates 2 μm. Fluorescence and transmission light intensities of representative single cells are measured longitudinally (dotted arrows). In the right plots, the intensities are shown in grey (transmission light), green (eGFP), red (mCherry), and blue (Hoechst33342) lines along the long axes of the cells. (C) Representative superresolution images of cells expressing Shi-eGFP with either ParA(wild-type)-mCherry, ParA(K15E)-mCherry or ParA(K15Q)-mCherry, or without ParA-mCherry. Fluorescence images are acquired at 4h after IPTG induction. Aberrant foci are indicated by white triangles. Scale bar indicates 1 μm. (D) Co-immunoprecipitation of ParA-mCherry with Shi-eGFP using GFP affinity beads. Cell extracts were prepared from strains used in (B). Whole cell (W), flow-through (F), and bead (B) fractions were analyzed by immunoblot using anti-RFP and anti-GFP antibodies. Control, cells expressing ParA and Shi without fluorescence fusions.

To investigate localization of ParA and Shi in the *P*. *putida* cell, we fused them individually with different fluorescent proteins. Induction of the two fusion proteins, ParA-mCherry and Shi-eGFP, from pME6032 by the addition of IPTG led to growth arrest and elongation of *P*. *putida* cells without ICE*clc* (Fig 2A and 2B), as observed in the previous experiments with wild-type ParA and Shi (S1 Fig) [14], demonstrating that the fusion proteins have comparable activities as the wild-type proteins. Regular epifluorescence microscopy showed globally distributed Shi-eGFP fluorescence within induced cells, while ParA-mCherry was colocalized with Hoechst 33342 fluorescence, a dye staining the nucleoid (Fig 2B). Cells visually appeared longer in case of co-expressing ParA-mCherry or Shi-eGFP, compared to each expressed individually (Fig 2A and 2B), but the subcellular localization of the proteins remained the same (Fig 2B). Superresolution imaging confirmed more clearly that wild-type ParA-mCherry colocalized with the nucleoid, whereas Shi-eGFP tended to localize to the cell membrane, further forming several distinctive foci (Fig 2C). These results suggested that ParA associates to chromosomal DNA, whereas Shi is a cytoplasmic protein with preference for the cell membrane.

We then examined whether the association of ParA to chromosomal DNA is indeed necessary for cell growth inhibition. Given that the ParA carries a predicted Walker ATPase motif, and that ATP binding and subsequent conformational change in the protein is known from other ParA-systems to be required for binding to DNA [19,20], we generated two ParA-mCherry mutants of which the N-terminal lysine 15 in the ATP-binding site (KGGVGKT) was substituted by either glutamine (K15E) or glutamic acid (K15Q). These mutations are expected to abolish ATP hydrolysis. Indeed, induction of the two ParA mutant proteins in conjunction with Shi-eGFP did not lead to cell growth inhibition, nor to elongated cells (Fig 2A and 2C). Interestingly, both ParA(K15E)-mCherry and ParA(K15Q)-mCherry fluorescence no longer colocalized with DNA, but generated aberrant unassociated local foci (Fig 2C). Also localization of Shi-eGFP in strains co-expressing the ParA mutants seemed to become more cytoplasmic and less membrane-enriched, while the membrane association of Shi-eGFP was still relatively obvious in the strain without ParA-mCherry (Fig 2C). These results thus show that mutations in ParA abolishing its complex formation with ATP alter its subcellular localization and that of Shi, which overrides the inhibitory effect on cell growth.

These results thus indicated that ParA and Shi act synergistically to inhibit growth of the host cell. One hypothesis for the synergistic action would be that ParA and Shi interact by protein-protein interactions (despite localizing broadly to different cell regions). To test this *in vivo*, we performed native co-immunoprecipitation using *P*. *putida* cells expressing ParA-mCherry and Shi-eGFP. Cell lysates were mixed with GFP-Trap beads, and bound proteins to the beads were detected by anti-GFP and anti-RFP antibodies. Immunoblots revealed enriched ParA-mCherry in the GFP-trapped fraction of Shi-eGFP, suggesting affinity of both proteins for each other (Fig 2D). However, a certain amount of ParA-mCherry was still detected in the flow-through fraction, suggesting that the interaction is dynamic, transient and reversible. This may explain why ParA-mCherry and Shi-eGFP appear mostly not colocalized in microscopy images.

### Dynamics of cellular growth inhibition by ParA and Shi

**Fig 3:**
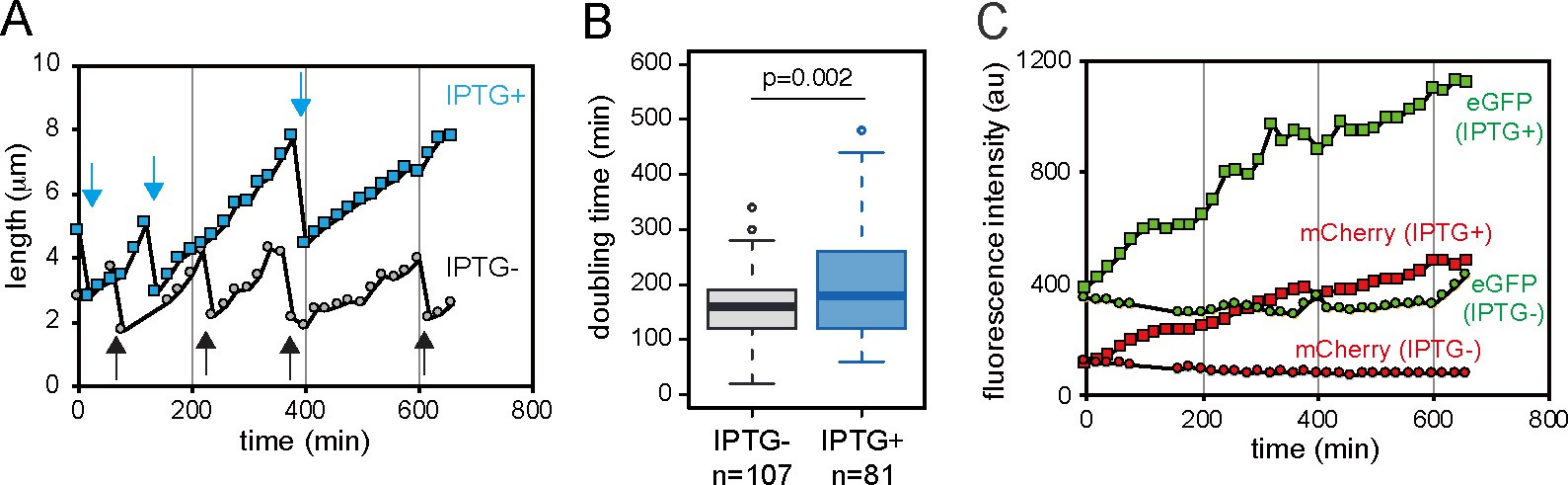
Dynamics of cellular growth and ParA and Shi expression at single-cell level of *P*. *putida*. (A) Cell length changes in two representative lineages with IPTG (leading to expression of ParA and Shi, blue squares) or without (grey circles). Cellular lengths measured at each time point connected for visibility with a line. Arrows indicate cell division events. (B) Box plots of doubling times in cells with or without IPTG induction. P-value in Wilcoxon rank test is indicated. (C) Average fluorescence intensity changes from Shi-eGFP (green) and ParA-mCherry (red) in individual cells in two representative lineages with (square) or without IPTG (circle). Same cell lineages as in panel A. Time indicates the duration from the start of the incubation.

**Fig 4:**
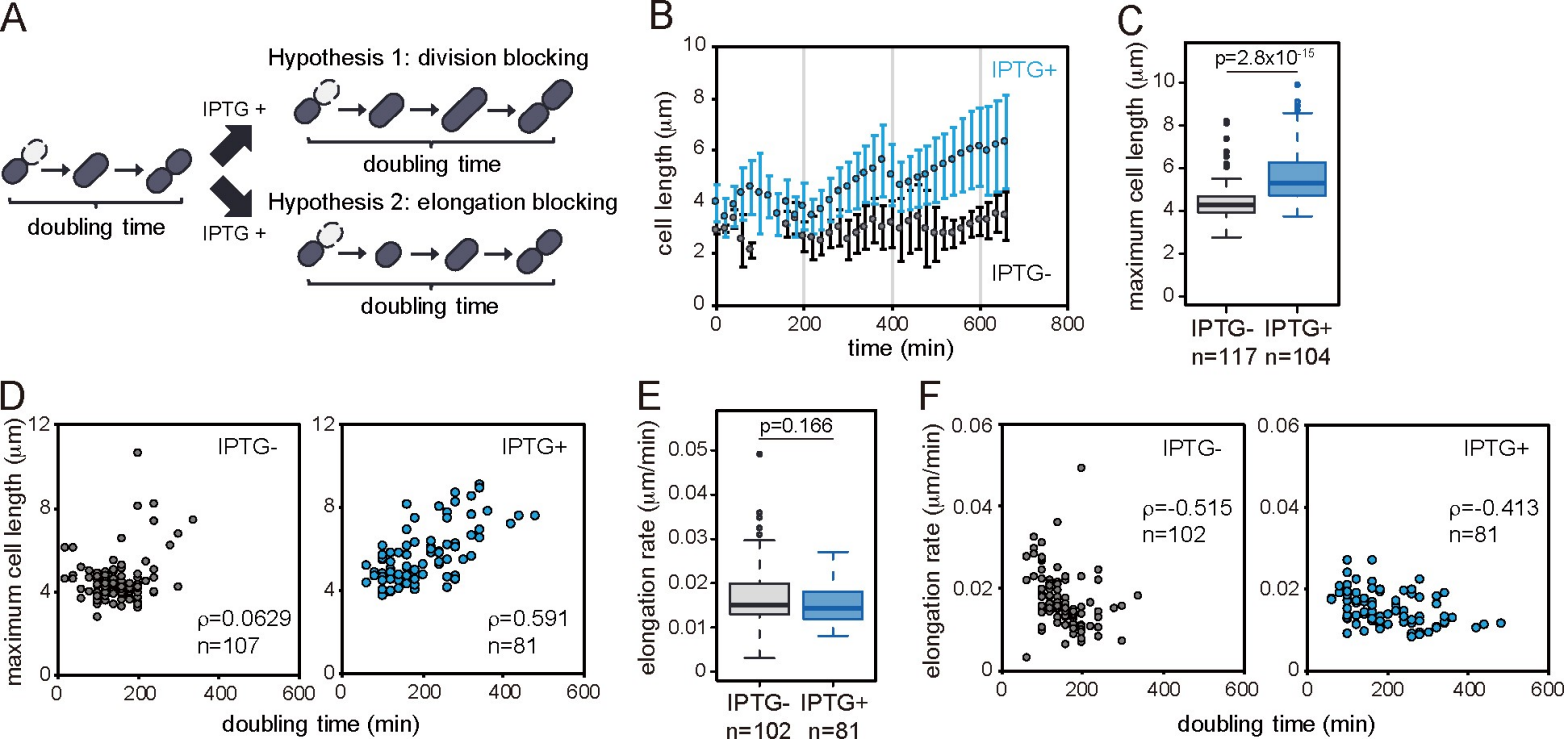
Experimental test of two working hypotheses for ParA-Shi-mediated cell growth inhibition. (A) Schematic illustrations of two scenarios of growth inhibition: division blocking (Hypothesis 1) or elongation blocking (Hypothesis 2). (B) Average cell length changes in individual cells of two representative micro colonies with IPTG (leading to ParA and Shi induction, blue circles) or without (grey circles). Average cell length at each time point calculated from all cells in the microcolony at that point. Error bars indicate standard deviations. (C) Maximum cell lengths across all cells incubated in presence or absence of IPTG. Cells which did not divide until the end of the experiments are eliminated from analysis. P-value in Wilcoxon rank test is indicated. (D) Correlation between maximum cell length and doubling time with (right panel) or without IPTG (left panel), plotted for all individual cells. Same datasets as presented in Fig 3B are used. Spearman’s correlation coefficient (*ρ*) and the number of cells used for analysis (n) are indicated. (E) Box plots of cellular elongation rate in the presence or absence of IPTG. Elongation rate of each cell is calculated by dividing the difference between start and final cell length with elapsed time. Cells which existed less than 4 frames (60 minutes) or did not divide until the end of the experiments are eliminated from analysis. P-value in Wilcoxon rank test is indicated. (F) Same as (D), but between elongation rate and doubling time.

To understand what might be the target mechanism of ParA and Shi to inhibit cell proliferation, we dynamically tracked cell elongation and division, and fluorescent protein expression in time-lapse microscopy. We used *P*. *putida* UWC1 expressing ParA-mCherry and Shi-eGFP under the control of LacI^q^/P_lac_ system, and monitored ~200 cells in the presence or absence of IPTG (S1 and S2 Movies). As expected from results shown in Fig 2A, induction of both proteins significantly increased the doubling time of individual cells (Fig 3). If the two proteins inhibit cell division itself, IPTG induction would increase cell length but not their elongation rate (Fig 4A, Hypothesis 1). In contrast, if they prevent the elongation process, its rate would decrease but the overall cell length before division would remain the same (Fig 4A, Hypothesis 2). To differentiate among the two hypotheses, we measured individual cell lengths and elongation rates in growing microcolonies in presence or absence of IPTG. Indeed, our results showed that the average cell length increased over time in presence of IPTG, whereas in the absence of IPTG it remained constant (Fig 4B and S3 Fig). Concomitantly, the average maximum length of the cell before division significantly increased in presence of IPTG, compared to that without IPTG (Fig 4C). Cellular doubling time and maximum cell length correlated strongly when ParA and Shi were co-expressed, whereas no correlation was observed without induction (Fig 4D). Production of the two proteins thus seems to lead to both an increase of the maximum cell length and its doubling time. In contrast, elongation rates of individual cells (as the difference of maximum cell length minus cell length at birth divided by doubling time) were not significantly different in cells exposed or not to IPTG (Fig 4E). Furthermore, negative correlations between elongation rate and doubling time were not significantly different in *P*. *putida* induced or not by IPTG (Fig 4F). These results thus support the hypothesis that ParA and Shi inhibit the cellular division rather than the elongation process.

### Effect of ParA and Shi on chromosome dynamics

**Fig 5:**
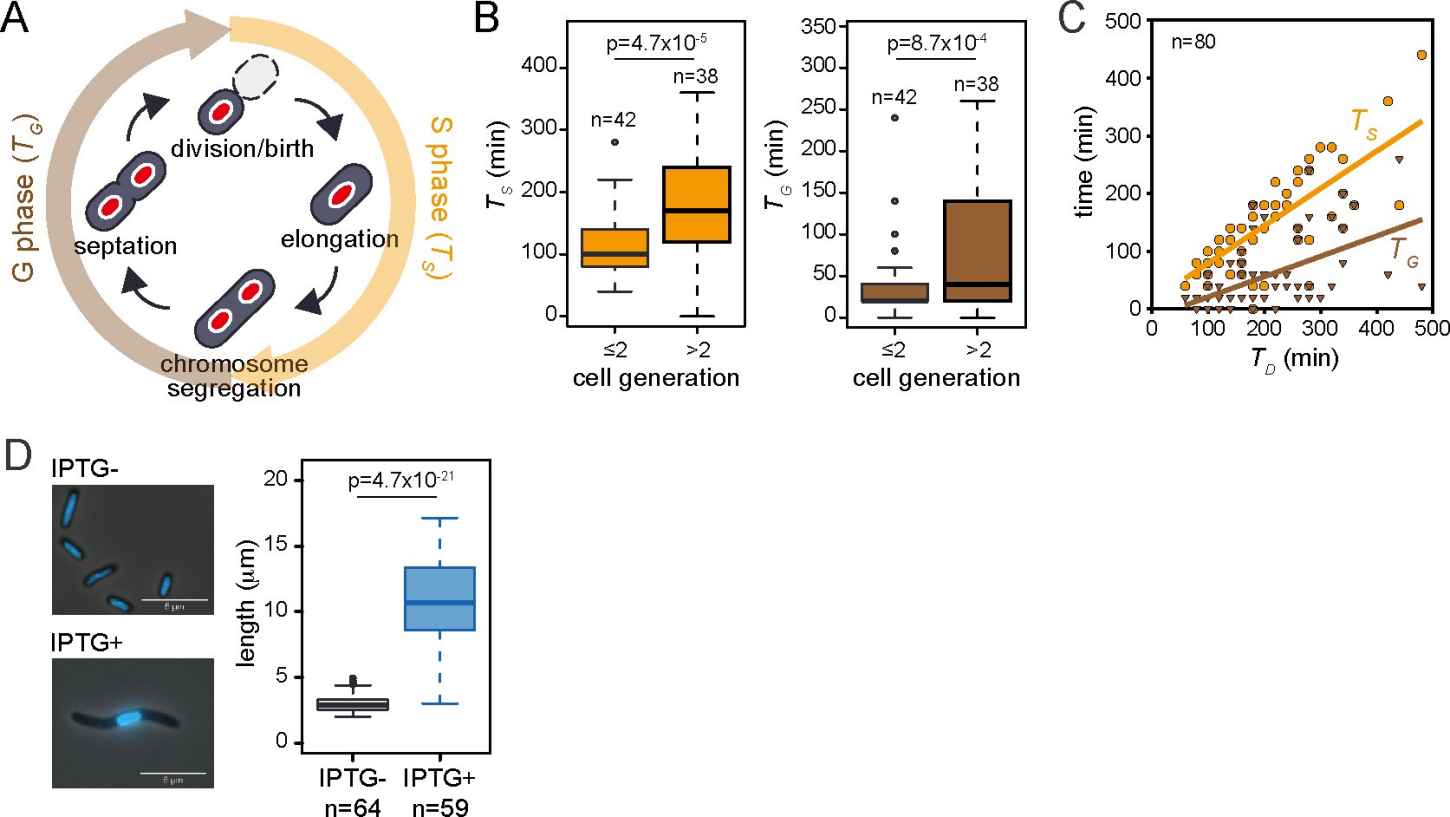
Effect of ParA and Shi on *P*. *putida* chromosome replication and segregation. (A) Schematic illustration of cell cycle of *P*. *putida*. S phase, elongation of newly emerged daughter cells, and chromosome replication and segregation (nucleoid shown as red oval). G phase is defined from nucleoid segregation to cell division. Time required for S and G phases are denoted as *T*_*S*_ and *T*_*G*_, respectively. Hence, the doubling time (*T*_*D*_) of the cell is the sum of *T*_*S*_ and *T*_*G*_. (B) Box plots of *T*_*S*_ (left) and *T*_*G*_ (right) of cells emerged in different time periods in the presence of IPTG. Here we regarded the cells pre-existing at the start of the time-lapse observation as the first generation. The doubling time of cells is not significantly changed by IPTG induction until the second division (S4 Fig), and thus we compared cells from the first and second generation with later offspring (>2 generations). P-value in Wilcoxon rank test is indicated. (C) Correlations of *T*_*S*_ or *T*_*G*_ with doubling time (*T*_*D*_) in the presence of IPTG. *T*_*S*_ (orange circle) and *T*_*G*_ (brown triangle) of 80 cells as (B) are plotted as function of their doubling time (*T*_*D*_). The linear regressions are estimated by MATLAB polyfit functions (*T*_*S*_ = 14.5 + 0.647*T*_*D*_, *T*_*G*_ = −14.5 + 0.353*T*_*D*_). The statistical significance of two slopes were tested by analysis of covariance using MATLAB function (p<0.001). (D) Representative micrographs (left panels) and box plots of cellular length (right) of single-nucleoid cells with or without IPTG. Merged images of phase-contrast and fluorescence (Hoechst33342) channels were acquired at 16h after IPTG induction. Scale bar indicates 5 μm. Asterisk indicates significance of difference (P<0.005) in Wilcoxon test.

As we found that ParA is a nucleoid-associated protein, we next investigated whether the inhibition of cell division by ParA and Shi occurs directly or indirectly via blocking chromosome replication/segregation in the S phase. If the proteins block the replication/segregation, it would take longer until the nucleoids can separate, which would result in an extended S phase and delayed cell division. We tracked ParA-mCherry localization to the nucleoid in proliferating *P*. *putida* cells which co-expressed Shi-eGFP, and calculated the doubling time of each individual cell (*T*_*D*_) as the sum of the time from cell birth to visible nucleoid separation (*T*_*S*_ in S phase) and the time between visible nucleoid segregation and cell division (*T*_*G*_ in G phase) (Fig 5A). We further compared *T*_*S*_ and *T*_*G*_ between the first generation offspring and later ones (>2 generation), to distinguish between cells that had not (generation 1) or had experienced IPTG (later generations, S4 Fig). Both *T*_*S*_ and *T*_*G*_ were significantly longer in ‘later’ than those of the ‘early’ cells (Fig 5B). *T*_*S*_ and *T*_*G*_ both positively correlated to the cellular doubling time, but the slope of *T*_*D*_-*T*_*S*_ in a linear regression model was significantly higher than that of *T*_*D*_-*T*_*G*_ (Fig 5C). These results indicate that, although both *T*_*S*_ and *T*_*G*_ increased by ParA and Shi expression, the increase of *T*_*S*_ has a greater contribution on the increased doubling time. In fact, given that the cellular elongation rate was not changed by ParA and Shi expression (Fig 4E), cells with a single nucleoid kept elongating without division (Fig 5D). These results strongly suggest that ParA and Shi act on extending the S phase.

### Consequence of growth inhibition by ParA and Shi

**Fig 6:**
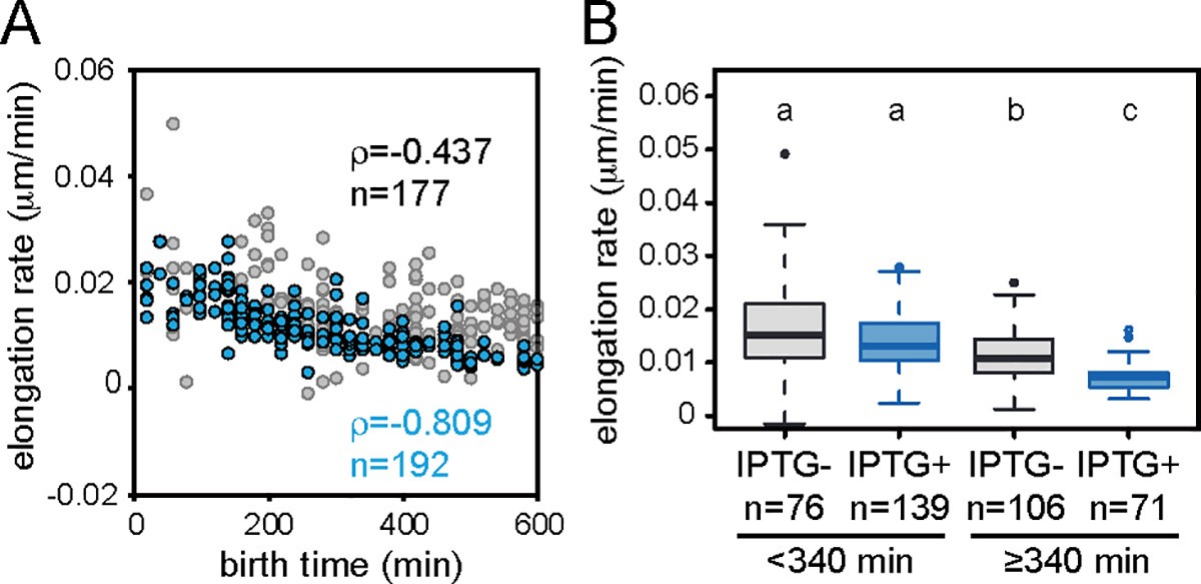
Cellular elongation rate depending on the birth time of cells. (A) Correlation between cellular elongation rate and birth time in the presence (blue) or absence (gray) of IPTG. (B) Box plots of cellular elongation rates in the different birth time of cells with (blue) or without IPTG (gray). Letters above plots show significance group based on Kruskal-Wallis test followed by Dwass-Steele-Critchlow-Fligner post hoc test (p<0.005).

Our results of time-lapse microscopy showed that the elongation rate of cells was not significantly changed by the ParA and Shi. If the elongation rate is constant without cell division, one would expect to observe extremely elongated cells during cultivation. However, cellular length plateaued at 3–4 times longer than that without ParA and Shi expression during overnight cultivation (Fig 5D). This result is in agreement with behavior of tc cells in the previous study: tc cells completely stop their growth after a few cell divisions [14]. We thus hypothesized that the elongation rate would also gradually decrease beyond a certain time point in cells. To test this hypothesis, we analyzed elongation rates of the cells observed in time-lapse microscopy as a function of the time they emerged (defined as birth time) in the microcolony. Elongation rates on average decreased over time regardless of the presence or absence of IPTG in the culture medium, but a stronger negative correlation was detected in the presence of IPTG (Fig 6A). Elongation rates were roughly indistinguishable between cells in presence or absence of IPTG during the first half of the experiment (<340 min, p = 0.0913), but significantly lower in incubations with IPTG during the second half (>340 min, p = 6.60 x 10^−6^) (Fig 6B). This suggests that elongation rates decrease in later generations of cellular offspring, consequently arresting the microcolony growth sooner than that of cells not induced for ParA and Shi.

### Prevalence of ParA and Shi in proteobacteria

**Fig 7:**
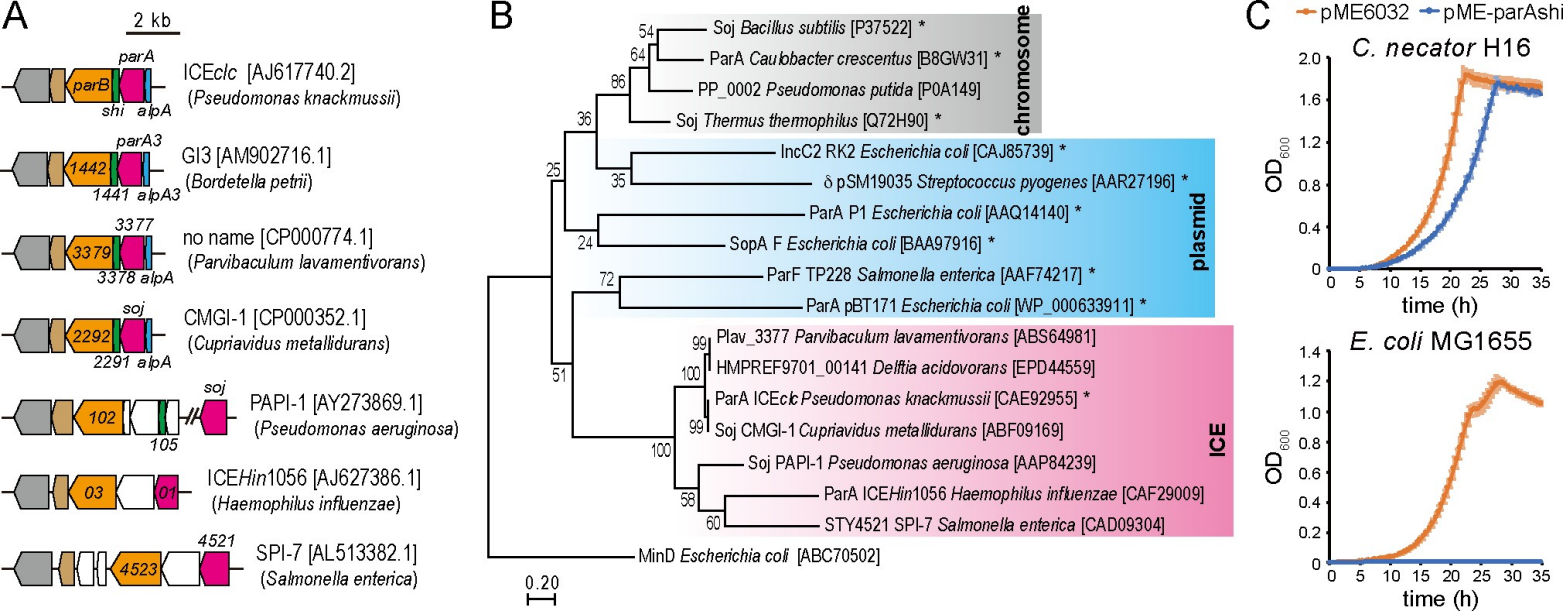
Phylogenetic analysis of ParA and effect of *parA*-*shi* expression in different bacteria. (A) Schematic illustration of *parA-shi* locus on various ICEs. ICE names (if named), species names and accession numbers are shown. Genes are indicated as in the respective genome accession. Coloration is based on predicted functions. (B) Maximum-likelihood (ML) tree based on the amino acid sequences of Walker ATPase family proteins. The 18 protein sequences are aligned and used for construction of the ML tree by using the Jones-Taylor-Thornton model. MinD is used as an outgroup. The bootstrap values (100 resampling) are shown on each branch. The tree is drawn to scale, with branch lengths measured in the number of substitutions per site. Proteins of which functions are experimentally demonstrated are denoted by asterisks. Accession numbers are shown in brackets. (C) Population growth of *C*. *necator* H16 (upper) and *E*. *coli* MG1655 (lower) cells carrying either pME6032 or pME-parAshi. Cells are cultured with IPTG, and their culture turbidity is measured. Error bars represent SD from the mean in triplicate assays.

The *parA*-*shi* locus is highly conserved in ICE*clc*-related genetic elements in other proteobacterial genomes, exceptions being ICE*Hin*1056 and SPI-7, which lack a *shi* homologue (Fig 7A). Phylogenetic analysis of Walker ATPases positioned ICE-ParA homologues into a single clade divergent from other ParA-family proteins involved in chromosome and plasmid partitioning (Fig 7B), suggesting that ICE-derived ParA proteins have diverged from other Walker ATPases specific for partitioning chromosomes or plasmids. On the other hand, Shi had been annotated as a hypothetical protein without any domains or motives presented in public databases at a statistically significant level (*E*<0.01). In the current database of NCBI, some hypothetical proteins that show <80% identities to Shi contain the HicA_toxin domain (pfam07927, *E*<0.01), a ribonuclease domain conserved in the HicA toxin of the type II toxin-antitoxin (TA) system [21]. Genes of those hypothetical proteins were found in various proteobacterial genomes, such as *Xhanthomonas*, *Pectobacterium*, *Pseudomonas*, and *Dickeya*, in the proximity of *parA*- and *parB*-like genes, while the *hicA* gene of the TA system is adjacent to its cognate *hicB* gene, encoding the anti-toxin protein. Despite the lack of apparent conserved domains, amino acid alignment and secondary structure prediction of those proteins suggest that Shi and its homologues share some conserved residues and the similar secondary structure with HicA proteins (S5A Fig). Phylogenetic analysis positioned Shi and its relatives into a different clade divergent from HicA toxins (S5B Fig). These results suggest that Shi is diverged from HicA of the type II TA system.

Given that ICE*clc*-related elements were distributed in a wide range of proteobacterial genomes, we examined the growth inhibitory effect of ParA-Shi in other bacterial genera. Induction of ParA and Shi from the plasmid pME6032 significantly inhibited the growth of both *Cupriavidus necator* H16 (beta-proteobacteria) and *E*. *coli* MG1655 (gamma-proteobacteria), although the effect was more moderate in *C*. *necator* H16 (Fig 7C). Despite repeated attempts to introduce the plasmids with and without *parA-shi* into *Sphingobium japonicum* UT26 (alpha-proteobacteria), no transformants were obtained when the plasmid contained *parA-shi*, probably due to leaky expression of ParA-Shi from the plasmid resulting in inhibition of the colony formation. These results suggest that ParA and Shi still exert inhibitory effect on cell growth of other proteobacteria.

## Discussion

While mobile DNA elements often provide selective advantage to host bacteria by conferring conditionally beneficial functions, such as antibiotic resistance, their presence and activity within the cell can be disadvantageous for the host. Additional physiological and energetic costs may arise from their replication, transcription and translation, as well as from horizontal transfer to the other hosts [22]. To minimize such costs and consequently increase fitness of mobile DNAs in a given ecological niche, they have evolved a variety of systems. Some plasmids encode H-NS-like proteins to silence derogative functions that may impede plasmid maintenance [23,24]. Another well-known system in plasmids is the genetic addiction by toxin-antitoxin (TA) genes, through which plasmid-free daughter cells accidentally emerging during cell proliferation are killed by toxin proteins that are more stable than antitoxins, assuming that such daughter cells can become competitors growing faster than cells still carrying plasmids [25]. Temperate bacteriophages express regulatory proteins, such as cI repressor in phage lambda, that not only stably maintain the lysogenic state in the host but also control host metabolic pathways to ensure host survival and efficient reproduction of bacteriophages [26–28]. As a system for ICEs, we have reported that ICE*clc* invokes a bistable decision between vertical and horizontal transmission [1]. Cells following the horizontal transmission pathway develop transfer competence, dedicated to transmit the ICE [17]. We show here how such tc cells additionally follow a pathway leading to cell growth arrest, which is induced by ICE*clc*-encoded ParA and Shi. Interestingly, the system of cell growth arrest is not absolute but gradual, which may have been selected for more efficient ICE transfer through small groups of tc cells [14]. There could be a trade-off between cell growth and ICE transfer, and thus the bistability (i.e., becoming tc cells at 3–5%) is apparently important to keep the balance.

The operon spanning from *alpA* to *parB* genes is highly conserved in other ICE*clc*-related elements found in many different proteobacteria, such as *Acidovorax*, *Burkholderia*, *Bordetella*, and *Pseudomonas* species including *P*. *aeruginosa* [12,13]. The first gene *alpA* is annotated as encoding a putative transcriptional regulator. AlpA was originally found in a cryptic P4-like prophage of *E*. *coli*, and acts as a positive transcriptional regulator of *slpA*, a P4 integrase gene [29]. AlpA expression results in the excision and loss of the cryptic prophage [30]. Curiously, ICE*clc* carries a P4-type integrase gene (*intB13*) that actually mediates its excision from the host chromosome [31]. These imply that both *intB13* and *alpA* of ICE*clc* may be derived from a P4-like phage ancestor and still maintain their regulatory relationship.

ParA and ParB encoded on ICEs exhibit significant homologies to those for chromosome or plasmid partitioning (*E*<10^−5^) (Fig 7). Partition is the most important system for stable segregation of those replicons to daughter cells. A class of the partition systems involves a specific DNA sequence on the segregating replicon that functions as the bacterial equivalent of centromere (e.g., *parS* site), and two proteins: one binds to the centromere (e.g., ParB) and the other is a Walker ATPase with non-specific DNA binding activity (e.g., ParA) [32,33]. Although the mechanism of the partition system with Walker ATPases is still under debate, recent studies more support the diffusion ratchet model in which ParB stimulates ATP hydrolysis of ParA, resulting in the destabilization of ParA-DNA binding and dynamic gradients of ParA-ATP complex as the driver of ParB-DNA cargo [34–36]. Using fluorescent fusion proteins and microscopic imaging, we here showed that ParA of ICE*clc* colocalizes with the chromosome of *P*. *putida* (Fig 2). Two amino acid substitutions we made at the ‘signature’ lysine residue of the Walker A motif in ParA, ParA(K15E) and ParA(K15Q), are supposed to interfere with the formation of ParA-ATP complex [20,37]. The former mutation prevents the protein from binding to ATP, whereas the latter still permits the binding but disturb the proper conformational change in the complex. We found that both mutants completely abolished the inhibitory effect on cell growth and the association with DNA (Fig 2), suggesting that ParA of ICE*clc* also causes its conformational change by binding to ATP, which is required for the growth inhibition. Considering such functional analogies, one could assume that the competition between ParA proteins from ICE*clc* and chromosome for DNA binding leads to the growth impairment. We revealed that ParA and Shi indeed inhibit chromosome replication/segregation, which consequently delays cell division (Fig 5). However, the mechanistic details may not be so simple, because ParA of ICE requires Shi but not ParB for the growth inhibition: the inhibitory effect is observed regardless of the presence or absence of ParB [14].

Shi is a curious protein in terms of its action and phylogenetic context. Protein sequences and phylogenetic analysis showed that, although some Shi homologues contain the HicA_toxin domain (pfam07927), they are obviously divergent from HicA toxins of the type II TA system (S5 Fig). HicA is an RNA interferase, causing mRNA and transfer mRNA degradation [38], and thus induction of HicA results in growth impairment of the host [38], whereas Shi did not when expressed alone (Fig 2A) [14]. We provided evidence for an *in vivo* physical interaction between ParA and Shi proteins, which further seemed to be transient or reversible (Fig 2D). Superresolution microscopy revealed further that Shi proteins are enriched in the cell membrane, but abolished when ParA is dysfunctional (Fig 2C). These characteristics suggest that Shi is functionally distinct from HicA. Taking into account the phylogeny of ParA and Shi, a possible hypothesis could be that both proteins anciently having different tasks in gram-negative bacteria, e.g., ParA for plasmid or chromosome partition and HicA for stress responses [21], got together in ICE*clc* or its relatives and modified their functions to help their horizontal transfer.

Our time-lapse imaging and quantitative analysis of cellular proliferation at the single-cell level enabled us to draw a several conclusions about the basic principles of growth impairment by ParA and Shi. First, our data indicate that ParA and Shi block cellular division rather than elongation (Fig 4). Cell division delay is likely the result of inhibition of chromosome replication/segregation (Fig 5). As ParA (Soj) of *Bacillus subtilis* inhibits DNA replication by interacting with DnaA, a replication initiator [39,40], ParA of ICEl*clc* is likely to act with a new partner outside of its original role in partitioning. Interestingly, however, also elongation rates become gradually longer in later generations from single mother cells forming microcolonies when ParA and Shi are expressed (Fig 6). This may point to some inherited aggravated effect of ParA and Shi in subsequent daughter cells, leading to on average smaller microcolonies than from cells in which ParA and Shi are not induced. As noted previously, the gradual arrest of cell division (as opposed to an immediate block of cell division in tc cells expressing ParA and Shi) was a selectable advantage for improved ICE*clc* (horizontal) fitness [17]. Further analysis is still needed to elucidate whether this effect is caused by direct actions of ParA and Shi or an indirect consequence of other cellular functions.

In conclusion, we provide here a new mechanism of cell growth impairment, exemplified by the ParA and Shi proteins of ICE*clc*. The action of ParA and Shi differs from known toxin-antitoxin systems and works as an adaptive strategy of ICE*clc* for increasing its horizontal transfer frequency and fitness.

## Materials and methods

### Bacterial strains and culture media

*Escherichia coli* DH5α (Gibco Life Technologies) and DH5α λ*pir* [17] for plasmid constructions was routinely grown at 37°C on LB medium [41]. *E*. *coli* MG1655 [42], *Cupriavidus necator* H16 [43], and *Pseudomonas putida* UWC1 [44] were cultured at 30°C on LB or type 21C minimal medium (MM) [45] containing either 10 mM Fructose or 5 mM 3-chlorobenzoate (3CBA). If necessary, antibiotics were added at the following concentrations; kanamycin 25 μg mL^-1^, gentamicin 20 μg mL^-1^, ampicillin 100 μg mL^-1^, and tetracycline 10 μg mL^-1^ for *E*. *coli*, 25 μg mL^-1^ for *C*. *necator* and 50 μg mL^-1^ for *P*. *putida*.

### DNA techniques

Preparation of plasmid and chromosomal DNAs, digestion with restriction endonucleases, DNA fragment recovery, DNA ligation, and transformation of *E*. *coli* cells were carried out according to established procedures [41] or to specific recommendations by the suppliers of the molecular biology reagents (Qiagen and Takara). The transformation and Tn*7* mutagenesis of bacterial cells by electroporation were performed as described previously [46]. Routine PCR was performed with ExTaq or PrimeStar DNA polymerase (Takara), and primers used are listed in S1 Table. All PCR products cloned were confirmed by sequencing with the BigDye Terminator version 3.1 (Applied Biosystems) and an ABI PRISM 3700 sequencer (Applied Biosystems).

### Plasmid constructions

Plasmids used in this study are listed in S2 Table. To introduce point mutations in *shi*, inverse PCR was carried out using pME-parAshi as a template with two different primer sets (140701 and 140702, or 140703 and 140704). Each PCR product was self-ligated, transformed in *E*. *coli*, and verified for correctness of the *parA*-*shi* locus with point mutations. To avoid PCR-based errors on the vector part, the 1.1-kb fragment containing *parA* and mutated *shi* genes was recovered from each plasmid by *Eco*RI-*Xho*I digestion, and recloned on a fresh pME6032.

To produce a C-terminal fusion of ParA to mCherry (i.e. ParA-mCherry), a ~900 bp fragment containing the *parA* gene without its stop codon was amplified using pME-parAshi as a template and primers (140801 and 140802). The fragment was cloned in *Eco*RI and *Hind*III sites on pBAM-link-mcherry [16], resulting in pBAM-parA-link-mcherry. A ~1.7-kb fragment including the *parA*-*mcherry* fusion gene was obtained by *Eco*RI-*Spe*I digestion of pBAM-parA-link-mcherry, subcloned into the same sites on pBluescriptIISK+, and then recovered by *Eco*RI-*Sac*I digestion. The fragment was cloned in the same sites on pME6032 to generate pME-parA-mcherry. To produce a C-terminal fusion of Shi to eGFP (i.e. Shi-eGFP), we first amplified a ~750 bp fragment containing the *egf*p gene using pJAMA23 [47] as a template and primers (140301 and 140302), in which the start codon of *egfp* was replaced by a short nucleotide sequence encoding 15 amino acids (KLPENSNVTRHRSAT) as a linker peptide. The fragment was cloned in *Hind*III and *Spe*I sites on pBAM-link-mcherry, resulting in pBAM-link-egfp. A ~280 bp fragment containing the *shi* gene without its stop codon was then amplified using pME-parAshi as a template and primers (140804 and 140805). The fragment was cloned in *Eco*RI and *Hind*III sites on pBAM-link-egfp, resulting in pBAM-shi-link-egfp. A ~1-kb of *Eco*RI-*Bgl*II fragment from pBAM-shi-link-egfp was cloned into *Eco*RI-*Bgl*II sites of pME6032 to generate pME-shi-egfp. The ~1.7-kb of *Eco*RI-*Spe*I fragment from pBAM-parA-link-mcherry and a ~1kb of *Xba*I-*Bgl*II fragment from pBAM-shi-link-egfp were together cloned in the *Eco*RI-*Bgl*II sites on pME6032 for generating pME-parA-mcherry-shi-egfp.

To fuse the *alpA* promoter with a promoter-less *egfp* gene, a ~750 bp fragment containing the *egfp* gene was first amplified using primers (150101 and 150102) and pJAMA23 [47] as a template. The fragment was cloned into the NsiI site on pUC18-mini-Tn7T-Gm [48], resulting in mini-Tn7T-egfp. A ~130 bp fragment containing the *alpA* promoter was then amplified using primers (190101 and 190102) and genomic DNA of *P*. *putida* carrying ICE*clc* as a template [49]. The fragment was introduced into BamHI and SpeI sites on mini-Tn7-egfp, consequently resulting in mini-Tn7T-PalpA-egfp.

### 5’ rapid amplification of cDNA ends (5’RACE)

Total RNA was isolated from *P*. *putida* clc6 cells grown in MM with 5 mM 3CBA until stationary phase, by using RNAprotect Bacteria Reagent and RNeasy Mini kit (Qiagen), following manufacture’s instruction. To remove contaminating genomic DNA, an 8 μg of the isolated RNA was further treated with 2U of TURBO DNase (Invitorgen) at 37°C for 1 h and purified with RNeasy spin columns (Qiagen). The amount of RNA was quantified with Qubit RNA BR assay kit (Invitrogen). A 500 ng of the RNA was used for the 5’RACE reaction with SMARTer RACE 5’/3’ kit (Clontech), according to manufacture’s instruction. In brief, a first-strand cDNA was synthesized by SMARTScribe Reverse Transcriptase, SMARTer II A oligonucleotide, and a specific primer 150201 that anneals the 5’ region of the *parB* gene. Using the cDNA as a template, the 1st 5’RACE PCR was performed with primers 150201 and Universal Primer Mix (UPM) provided with the kit. To increase specificity, the 2nd PCR was carried out with the 1st PCR product as a template using primers 140705 and short UPM provided with the kit. The 2nd PCR product (1.4 kb) was purified from an agarose gel and cloned into the provided pRACE vector. The plasmid carrying the 5’RACE product was sequenced with M13.R primer to determine the transcription starting site of *parA* and *shi* genes.

### Bacterial growth test in liquid media

Bacterial strains were pregrown in MM with fructose and tetracycline until stationary phase and adjusted to OD_600_ = 3.0. The preculture was reinoculated with 0.1% dilution into the fresh medium containing 1 mM IPTG. Optical density (OD_600_) was measured every 0.5 h, and its mean and standard deviation were calculated by biological triplicates.

### Microscopy

*P*. *putida* UWC1 derivatives were precultured in MM with fructose until stationary phase and then diluted 1% into the fresh medium containing 1 mM IPTG. After 4 h incubation, cells were stained by Hoechst33342. Cells and fluorescent proteins were imaged with a Zeiss Axio Observer epifluorescence microscope (Carl Zeiss). Images were taken with a Axiocam 506 monochrome camera (Carl Zeiss), a 100x/1.40 oil immersion Plan-Apochromat lens (Carl Zeiss) at exposure times of 350 ms for phase contrast and 100 ms for fluorescence images. The light source and filter used for fluorescence imaging was Zeiss Colibri7 and Filter Set 81 HE, respectively. Images were digitally recorded as 16-bit TIFF-files using the Zeiss Zen software, and analyzed using METAMORPH (Molecular Devices). Super-resolution images were observed using a LSM800 confocal laser scanning microscope, equipped with a 100x/1.46 oil immersion alpha Plan-Apochromat lens and an Airyscan detector (Carl Zeiss). mCherry, eGFP, and Hoechst33342 were excited with 561 nm, 488 nm, and 405nm lasers, respectively. Airyscan processing was performed with the 2D SR mode. Images for display were artificially colored ‘red’ (for mCherry), ‘green’ (for eGFP), or ‘blue’ (for Hoechst 33342), and then auto-leveled and cropped to the final resolution and image size using Adobe Photoshop (Adobe Inc.).

### Time-lapse experiments

*P*. *putida* cells containing the pME-parA-mcherry-shi-egfp plasmid were pre-cultured in LB medium supplemented with tetracycline for over 16 h. The culture was diluted to the density of OD_600_ = 0.04 with MM plus 10 mM fructose and tetracycline. A 10 μL of the diluted culture was plated onto 1.0% agarose pad of the MM in the closed cultivation chamber (H. Saur Laborbedarf) [14]. The chamber was connected to a syringe by 1x2 mm silicone tubes, and filled with the liquid MM with or without 0.2 mM IPTG by CX07100 syringe pump (Isis, Japan) on the beginning of the experiments. Time-lapse measurements of the cellular growth in the closed cultivation chamber were performed using a Zeiss Axio Observer microscope with ZEN software (Carl Zeiss). Stabilization of Z-offset in each position were facilitated by the use of Definite Focus (Carl Zeiss) during the experiments. The cultivation chamber and the microscope were kept at 30°C in an acrylic box with heater unit (Tokken, Japan) during the time-lapse experiment. Images were acquired with an Axiocam 506 mono and a 100x/1.40 oil lens with Colibri LED excitation light source (Carl Zeiss). The cells were exposed for 100 ms using 475nm and 555nm, both at 15% power, for taking eGFP and mCherry fluorescence images, respectively. For the acquisition of phase contrast images, transillumination LED light was irradiated to the cells for 100 ms at 3.8 V power. The time-lapse interval was 20 min. For the data analysis, we used the images acquired after 60 min of the experiments to wait for the temperature and the culture condition in the chamber to be stable.

### Image processing and parameterization of cellular characteristics

Five (IPTG−) and 11 micro-colonies (IPTG+) on the agarose pad were randomly chosen for analysis. Firstly, we processed the microscopy images using ImageJ (NIH) for subtracting background signals and enhancing the signals from the region of the cells. Based on these processed images, we made binary mask images by detecting cell contours, using Schnitzcells [50], a MATLAB based software (MathWorks, USA). In IPTG- conditions, we used the processed phase-contrast images for making masks. For generating the mask images in IPTG+ conditions, we used eGFP fluorescence images because the eGFP fluorescence distributed in cytoplasm region and facilitated clear detection of cell boundaries. Using these mask images, we tracked individual cell across multiple images by Schnitzcells. Finally, we obtained the several information for individual cell such as duration, transition of cell length, division (birth) time, change of eGFP and mCherry mean fluorescence intensity and daughter-parent relation. These data were further processed as necessary.

### Cell lysate preparation and native co-immunoprecipitation

*P*. *putida* UWC1 cells carrying pME6032 derivatives were precultured in MM with fructose and tetracycline until stationary phase and then diluted 1% into the fresh medium containing 1 mM IPTG. After 4 h incubation, cells were washed twice with 1 ml of PBS and suspended in 500 μl of the TENG buffer (50 mM Tris-HCl, pH7.5; 0.5 mM EDTA; 150 mM NaCl; 5% glycerol; 1% ProteoGuard EDTA-Free Protease Inhibitor Cocktail (Takara)). Cell lysates were subsequently extracted by sonication, and their protein concentrations were measured using Protein Assay kit (BIO-RAD). The lysate was diluted up to 400 μg of total protein in 500 μl of the ice-cold dilution buffer (50 mM Tris-HCl, pH7.5; 150 mM NaCl; 0.5 mM EDTA) and used as a whole cell fraction. The diluted lysate was mixed with Blocked Agarose Beads (chromotek) for 1 h at 4°C, to remove non-specific proteins bound to agarose beads. After centrifugation at 2,500x g for 2 min, the supernatant was mixed with GFP-Trap_A beads (chromotek) for 3 h at 4°C. After centrifugation, while the supernatant was used as a flow-through unbound fraction, the beads were gently washed with the ice-cold dilution buffer and resuspended with SDS sample buffer. Resuspended beads were boiled and centrifuged to extract proteins bound to the beads. Three fractions (i.e. whole cell, flow-through, and beads) extracted from each strain were used for western blot analysis.

### Western blot analysis

Cell lysates and fractions containing proteins were subjected to 12.5% SDS-PAGE analysis and immunoblotting. Rabbit polyclonal antibody to GFP (1:1000, MBL) and rabbit polyclonal antibody to RFP (1:1000, MBL), were used as primary antibodies, whereas horseradish peroxidase–conjugated antibody to rabbit (1:1000, Cell Signaling) was used as secondary antibodies. Immobilon Western (Millipore) was used for detection. Images were captured with ChemidocTM XRS+ systems (BIO-RAD).

### Phylogenetic analysis

The amino acid sequences of Walker ATPases involved in either chromosome or plasmid partitioning and those orthologous encoded on ICEs were obtained from the NCBI GenBank. The sequences were aligned with the program MUSCLE (https://www.ebi.ac.uk/Tools/msa/muscle/), and the Maximum-likelihood (ML) tree was then reconstructed using MEGA 7.0.26 with Jones-Taylor-Thornton model, Nearest-Neighbor-Interchange (NNI) move and 100 bootstrap replicates [51]. A discrete Gamma distribution was used to model evolutionary rate differences among sites. All positions containing gaps and missing data were eliminated. There were a total of 185 positions in the final dataset. The tree was rooted with MinD, another Walker ATPase localized on the cellular membrane, from *E*. *coli* MG1655. Alignment of the amino acid sequences of Shi from ICEs with close hits in the GenBank nr database and construction of the tree were performed with the same procedure described above. The tree was generated using a total of 49 positions and rooted with MazF, another type II toxin with different secondary structure from HicA, from *E*. *coli* MG1655.

## Supporting information

S1 Fig Effect on population growth of different point mutations in *shi*. *P*. *putida* UWC1 cells carrying pME6032 with different *parA*-*shi* fragments are cultured with IPTG, and their culture turbidity is measured. Error bars represent standard deviation (SD) from the mean in triplicate assays.
(TIF)

S2 Fig Expression of the P_alp_ promoter in single cells of *P*. *putida*. (A) Normal quantile-quantile plots showing distribution of eGFP (from P_alp_) fluorescence intensities at single-cell levels in the stationary phase populations of UWC1 carrying ICE*clc*. Cutoff (dashed line) between P_alp_-active (tc) and -inactive (non-tc) cells was calculated according to Reinhard and van der Meer [52]. Median (horizontal line), Minimum, and Maximum (vertical lines) fluorescence values of the inactive population are indicated, and the percentage and the mean fluorescence value of the active population are described. (B) Bar plots showing mean proportions of tc cells in UWC1 carrying wild-type ICE*clc* (WT) or *shi*-deleted ICE*clc* (Δ*shi*). The percentages were calculated with the same method as (A), based on the eGFP (from P_inR_) fluorescence intensities at single-cell levels in the stationary phase populations. Mean proportions and their standard deviations were calculated from three biological replicates, each of which includes more than 1,000 cells. P-value in two-tailed t test is indicated. (C) Box plots showing eGFP expression levels from P_tac_ promoter on pME6032 after 4h induction in UWC1 or P_alp_ promoter on mini-Tn*7* in stationary phase of UWC1 carrying ICE*clc*. Tc and non-tc cells are distinguished according to (A).
(TIF)

S3 Fig Example of transitions of mean cell length in single colonies with or without IPTG induction. At each time point, average cell length of *P*. *putida* are calculated for total cells in the colony. Error bars indicates standard deviations. Colony i1-i3, with IPTG; Colony n1-n3, without IPTG.
(TIF)

S4 Fig Box plots showing doubling time of cells emerged in different time periods under the IPTG induction. Note that the doubling time of the cells less than the second generation was statistically indistinguishable from that without IPTG, while that over second generation was significantly increased. P-values of pairwise comparisons were indicated, based on Kruskal-Wallis test followed by Dwass-Steele-Critchlow-Fligner post hoc test.
(TIF)

S5 Fig Phylogeny of HicA and Shi-like proteins. (A) Alignment of the amino acid sequences of nine HicA proteins found in *hicA-hicB* locus and nine Shi-like proteins found in *parA-shi-parB* locus of various bacterial genomes. Sequences are denoted by their GenBank Accession numbers and protein names or locus tags. The last two HicA proteins, of which crystal structures have been resolved, are also denoted by their PDB Accession numbers, and their secondary structures are shown below in italics: H and E indicate alpha-helix and beta-sheet, respectively. Predicted secondary structure of Shi via JPred (http://www.compbio.dundee.ac.uk/jpred4/index.html) is shown above. Positions with identical amino acids are enclosed. Conserved hydrophobic, polar, and positively charged residues are highlighted in green, purple, and blue, respectively. (B) Maximum-likelihood (ML) tree based on the alignment of (A) with MazF, another type II toxin, used as an outgroup. The ML tree was constructed using the Jones-Taylor-Thornton model. The bootstrap values are shown on each branch. The tree is drawn to scale, with branch lengths measured in the number of substitutions per site. Proteins which contain the HicA_toxin domain (pfam07927, *E*<0.01) are denoted by asterisks.
(TIF)

S1 Movie Time-lapse movie of *P*. *putida* UWC1 carrying pME-parA-mcherry-shi-egfp, cultured on agarose-pad without IPTG induction. (AVI)

S2 Movie Time-lapse movie of *P*. *putida* UWC1 carrying pME-parA-mcherry-shi-egfp, cultured on agarose-pad with IPTG. (AVI)

S1 Table Oligonucleotides used for PCR amplification. (DOCX)

S2 Table Plasmids used in this study. (DOCX)
