# Supplementary figures and images for: A novel system of bacterial cell division arrest implicated in horizontal transmission of an integrative and conjugative element
Source: PLoS Genet. 2019 Oct 14;15(10):e1008445. doi: 10.1371/journal.pgen.1008445 (PMC6812849; doi:10.1371/journal.pgen.1008445)

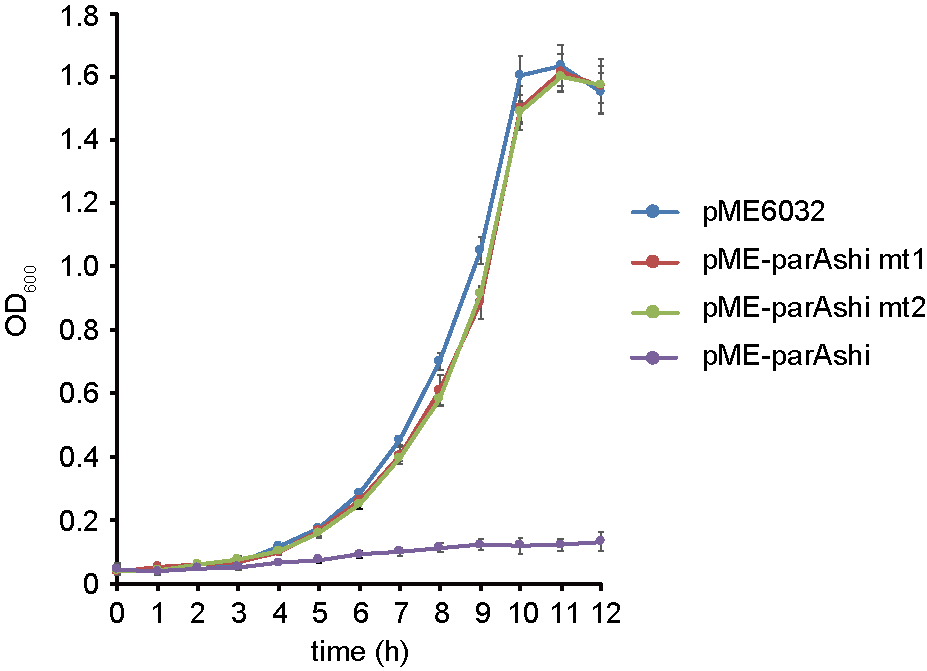

Supplement: S1 Fig — P. putida UWC1 cells carrying pME6032 with different parA-shi fragments are cultured with IPTG, and their culture turbidity is measured. Error bars represent standard deviation (SD) from the mean in triplicate assays. (TIF) [file pgen.1008445.s001.tif]

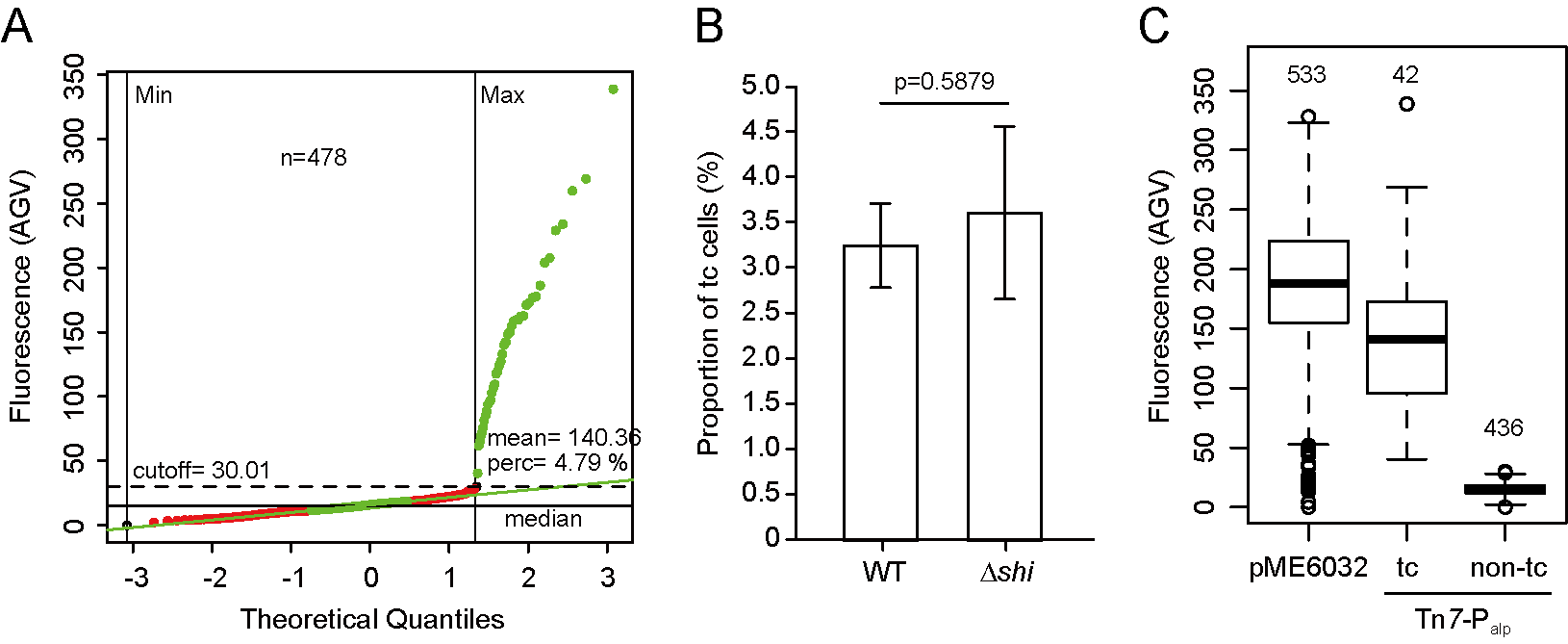

Supplement: S2 Fig — (A) Normal quantile-quantile plots showing distribution of eGFP (from Palp) fluorescence intensities at single-cell levels in the stationary phase populations of UWC1 carrying ICEclc. Cutoff (dashed line) between Palp-active (tc) and -inactive (non-tc) cells was calculated according to Reinhard and van der Meer [52]. Median (horizontal line), Minimum, and Maximum (vertical lines) fluorescence values of the inactive population are indicated, and the percentage and the mean fluorescence value of the active population are described. (B) Bar plots showing mean proportions of tc cells in UWC1 carrying wild-type ICEclc (WT) or shi-deleted ICEclc (Δshi). The percentages were calculated with the same method as (A), based on the eGFP (from PinR) fluorescence intensities at single-cell levels in the stationary phase populations. Mean proportions and their standard deviations were calculated from three biological replicates, each of which includes more than 1,000 cells. P-value in two-tailed t test is indicated. (C) Box plots showing eGFP expression levels from Ptac promoter on pME6032 after 4h induction in UWC1 or Palp promoter on mini-Tn7 in stationary phase of UWC1 carrying ICEclc. Tc and non-tc cells are distinguished according to (A). (TIF) [file pgen.1008445.s002.tif]

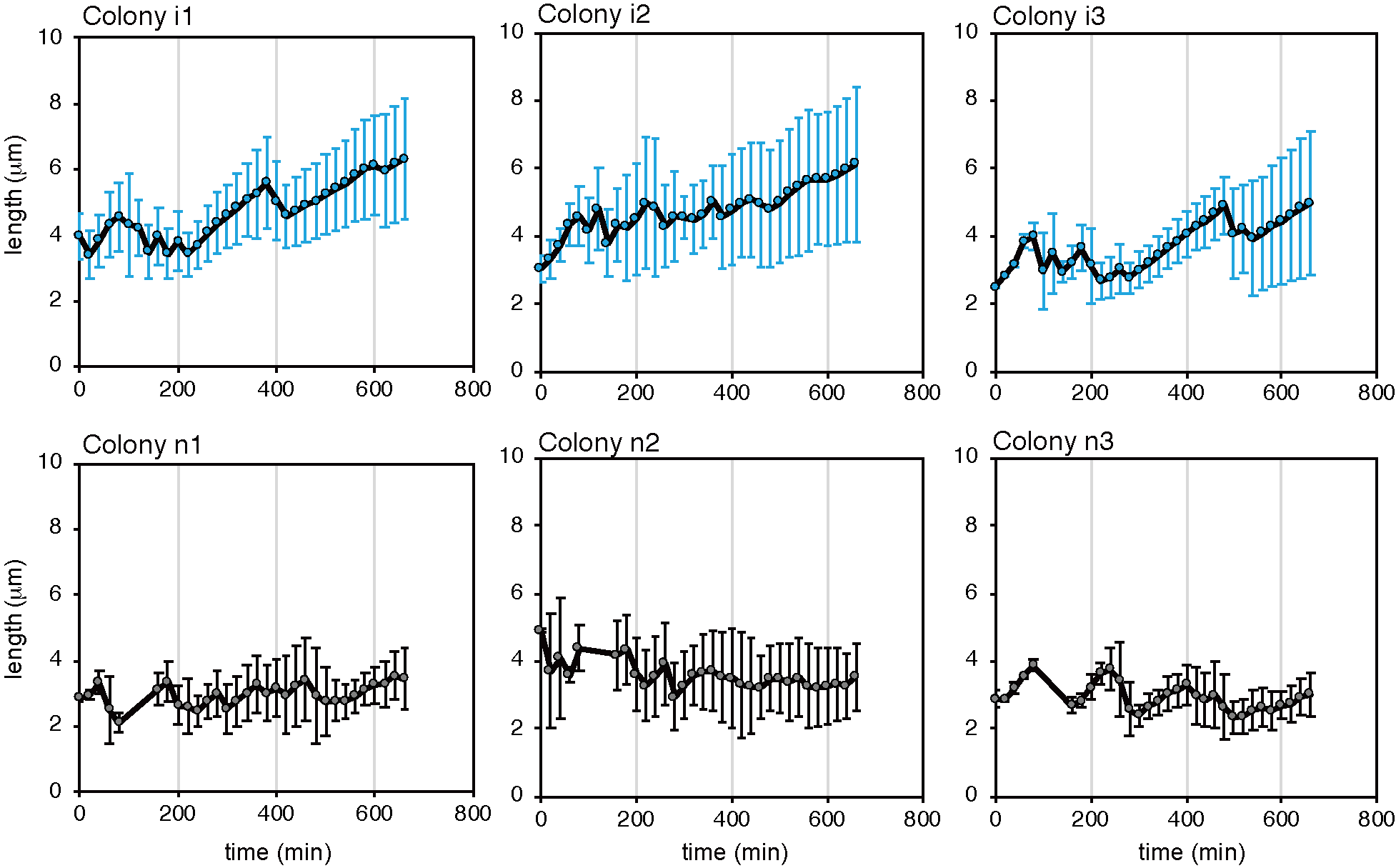

Supplement: S3 Fig — At each time point, average cell length of P. putida are calculated for total cells in the colony. Error bars indicates standard deviations. Colony i1-i3, with IPTG; Colony n1-n3, without IPTG. (TIF) [file pgen.1008445.s003.tif]

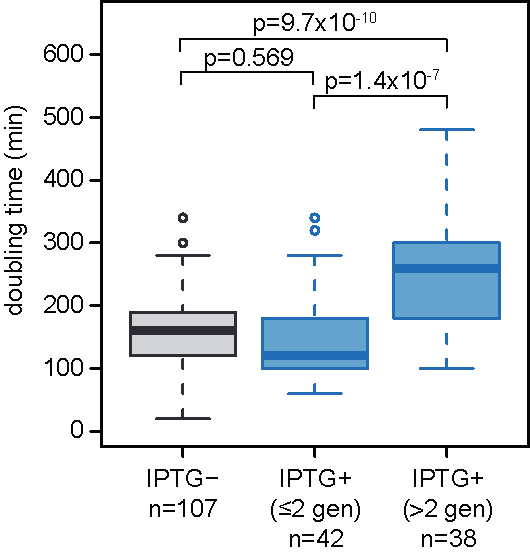

Supplement: S4 Fig — Note that the doubling time of the cells less than the second generation was statistically indistinguishable from that without IPTG, while that over second generation was significantly increased. P-values of pairwise comparisons were indicated, based on Kruskal-Wallis test followed by Dwass-Steele-Critchlow-Fligner post hoc test. (TIF) [file pgen.1008445.s004.tif]

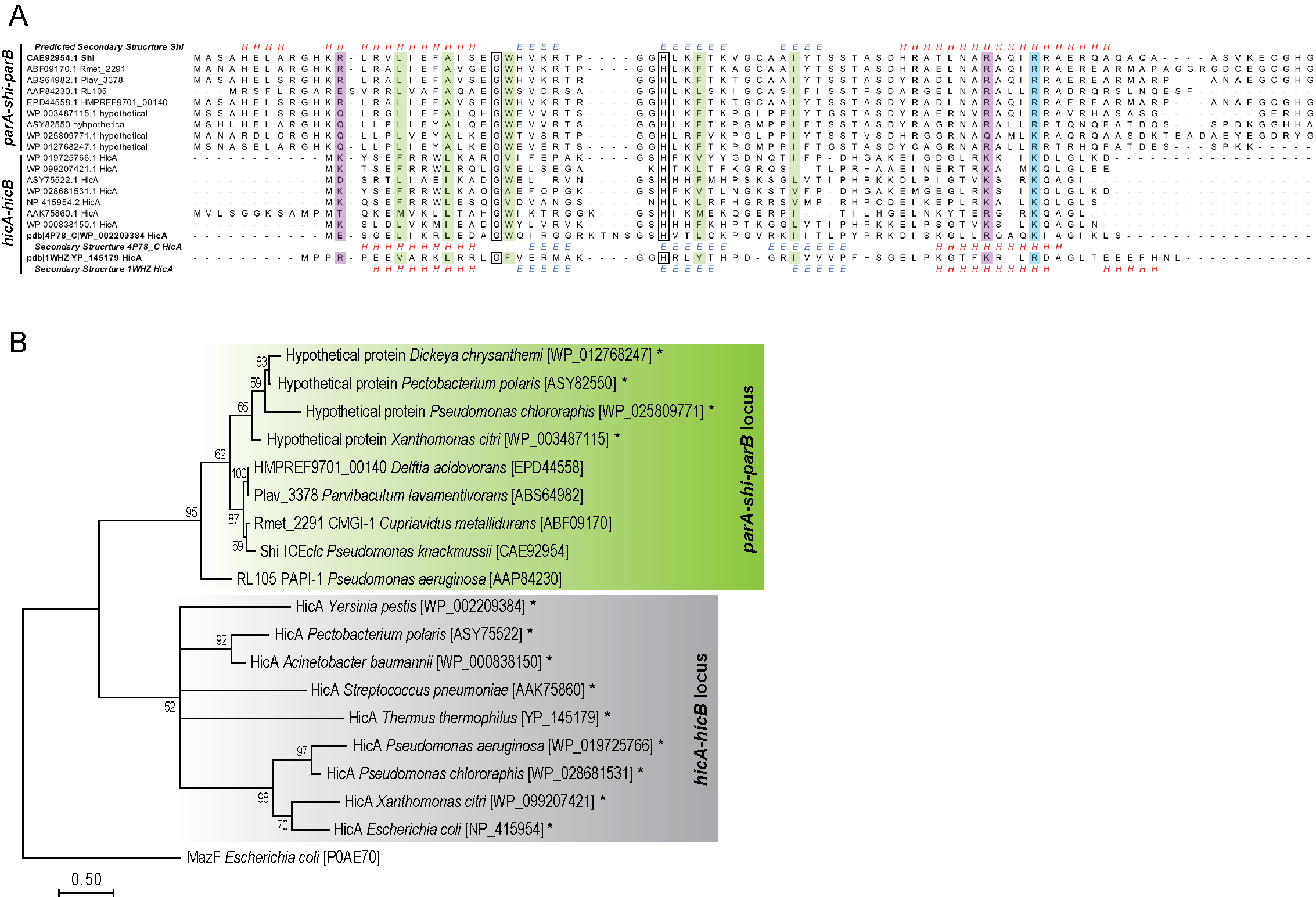

Supplement: S5 Fig — (A) Alignment of the amino acid sequences of nine HicA proteins found in hicA-hicB locus and nine Shi-like proteins found in parA-shi-parB locus of various bacterial genomes. Sequences are denoted by their GenBank Accession numbers and protein names or locus tags. The last two HicA proteins, of which crystal structures have been resolved, are also denoted by their PDB Accession numbers, and their secondary structures are shown below in italics: H and E indicate alpha-helix and beta-sheet, respectively. Predicted secondary structure of Shi via JPred (http://www.compbio.dundee.ac.uk/jpred4/index.html) is shown above. Positions with identical amino acids are enclosed. Conserved hydrophobic, polar, and positively charged residues are highlighted in green, purple, and blue, respectively. (B) Maximum-likelihood (ML) tree based on the alignment of (A) with MazF, another type II toxin, used as an outgroup. The ML tree was constructed using the Jones-Taylor-Thornton model. The bootstrap values are shown on each branch. The tree is drawn to scale, with branch lengths measured in the number of substitutions per site. Proteins which contain the HicA_toxin domain (pfam07927, E<0.01) are denoted by asterisks. (TIF) [file pgen.1008445.s005.tif]
