# Supplementary material for: A novel system of bacterial cell division arrest implicated in horizontal transmission of an integrative and conjugative element
Source: PLoS Genet. 2019 Oct 14;15(10):e1008445. doi: 10.1371/journal.pgen.1008445 (PMC6812849; doi:10.1371/journal.pgen.1008445)
Supplement: S1 Table — (DOCX) [file pgen.1008445.s008.docx]

**Table S1.** Oligonucleotides used for PCR amplification.

| Primer number | Sequence 5’-3’ | Purpose |
| --- | --- | --- |
| 150201 | CACGGATGGAAGCCTTGATTTC | 5’RACE |
| 140705 | TCAGCCATGGCCGCATTC | 5’RACE |
| 120502 | GATCCTTGGAGCCCTTGC | Amplify and sequence multiple cloning sites on pME6032 |
| 120503 | ACGGTTCTGGCAAATATTCTG | Amplify and sequence multiple cloning sites on pME6032 |
| 140701 | GCTGCCTCCCAGTCTCAG | Inverse PCR with pME-parAshi |
| 140702 | ACGGCAAGCGCACATGAAC | Inverse PCR with pME-parAshi, adding a point mutation at ATG of *shi* (to ACG) |
| 140703 | TTCATGTGCGCTTGCCATG | Inverse PCR with pME-parAshi |
| 140704 | TGAGCCCGCGGCCACAAGCG | Inverse PCR with pME-parAshi, substituting L7 of *shi* to TGA (opal mutation) |
| 140301 | AAGCTTccggaaaattcgaacgttacgcgtcaccggtcggccaccAGTAAAGGAGAAGAACTTTTCACTG | Amplify ATG-less *egfp* with a poly-linker sequence |
| 140302 | ACTAGTAGATCTATTTGTATAGTTCATCCATGCCAT | Amplify ATG-less *egfp* |
| 140801 | tttAAGCTTTGTGCGCTTGCCATGCTG | Amplify *parA* gene without its stop codon |
| 150101 | CCACTAGTGAGCTCATGCATCTGATTAACTTTATAAGGAGGA | Amplify *egfp* gene |
| 150102 | AAGCTAATTCGATCATGCATGCGGCCGCTATTTGTATAGTTCATCCATGC | Amplify *egfp gene* |
| 140802 | tttGAATTCCCTATCCCTAGACGGAGA | Amplify *parA* gene |
| 140803 | tttAAGCTTGCCATGGCCGCATTCCTTTAC | Amplify *shi* gene |
| 140804 | tttTCTAGACCTTCTGAGACTGGGAGGCAG | Amplify *shi* gene without its stop codon |
| 170903 | TTCGCCGACGCCGCCCTTGG | inverse PCR to make K15E mutation of *parA* on pBAM-parA-link-mcherry |
| 170904 | TTGGCCGACGCCGCCCTTGG | inverse PCR to make K15Q mutation of *parA* on pBAM-parA-link-mcherry |
| 170905 | ACGACCACCGCAGCCAACCTC | counterpart for 170903 and 170904 |
| 190101 | GCAGCCCGGGGGATCCTGATATTTGCAAGCCCGAATC | Amplify *alpA* promoter |
| 190102 | GCATGAGCTCACTAGTGGCGGTCACTCCTGAACG | Amplify *alpA* promoter |
