# Supplementary material for: A novel system of bacterial cell division arrest implicated in horizontal transmission of an integrative and conjugative element
Source: PLoS Genet. 2019 Oct 14;15(10):e1008445. doi: 10.1371/journal.pgen.1008445 (PMC6812849; doi:10.1371/journal.pgen.1008445)
Supplement: S2 Table — (DOCX) [file pgen.1008445.s009.docx]

**Table S2.** Plasmids used in this study.

| Name | Descriptions | Reference |
| --- | --- | --- |
| pME6032 | pVS1-p15A ori shuttle vector carrying *lacI*^q^-P_tac_; Tc^R^ | [1] |
| pME-parAshi | pME6032 derivative carrying *parA* and *shi* | [2] |
| pME-parA | pME6032 derivative carrying *parA* | [2] |
| pME-shi | pME6032 derivative carrying *shi* | [2] |
| pME-egfp | pME6032 derivative carrying *egfp* | This study |
| pUX-BF13 | R6K ori, source of Tn7 transposase; Ap^R^ | [3] |
| pUC18-mini-Tn7T-Gm | pUCori mini-Tn*7* vector; Gm^R^ | [4] |
| mini-Tn7T-egfp | pUC18-mini-Tn7T-Gm derivative carrying promoter-less *egfp* | This study |
| mini-Tn7T-PalpA-egfp | mini-Tn7T-egfp derivative carrying *alpA* promoter | This study |
| mini-Tn*5*-PinR-echerry | mini-Tn*5* carrying *inrR* promoter fused with *echerry* | [5] |
| mini-Tn*5*-PinR-egfp | mini-Tn*5* carrying *inrR* promoter fused with *egfp* | [5] |
| pME-parAshi_mt1 | pME6032 carrying *parA* and mutated *shi* (substitute ATG to ACG) | This study |
| pME-parAshi_mt2 | pME6032 carrying *parA* and mutated *shi* (substitute CTG to TGA) | This study |
| pBAM-link-mcherry | R6Kori mini-Tn*5* vector for C-terminal mCherry fusion | [5] |
| pBAM-link-egfp | R6Kori mini-Tn*5* vector for C-terminal eGFP fusion | This study |
| pBAM-parA-link-mcherry | pBAM-link-mcherry carrying *parA* | This study |
| pBAM-shi-link-egfp | pBAM-link-egfp carrying *shi* | This study |
| pME-parA-mcherry | pME6032 carrying *parA*-*mcherry* translational fusion | This study |
| pME-shi-egfp | pME6032 carrying *shi-egfp* translational fusion | This study |
| pME-parA-mcherry-shi-egfp | pME6032 carrying *parA*-*mcherry* and *shi-egfp* translational fusions | This study |
| pME-parA(K15E)-mcherry-shi-egfp | pME6032 carrying *parA*(K15E)-*mcherry* and *shi-egfp* translational fusions | This study |
| pME-parA(K15Q)-mcherry-shi-egfp | pME6032 carrying *parA*(K15Q)-*mcherry* and *shi-egfp* translational fusions | This study |

Reference:

1. Heeb S, Blumer C, Haas D. Regulatory RNA as mediator in GacA/RsmA-dependent global control of exoproduct formation in Pseudomonas fluorescens CHA0. J Bacteriol. 2002;184(4):1046-1056.

2. Reinhard F, Miyazaki R, Pradervand N, van der Meer JR. Cell differentiation to "mating bodies" induced by an integrating and conjugative element in free-living bacteria. Curr Biol. 2013;23(3):255-259.

3. Koch B, Jensen LE, Nybroe O. A panel of Tn7-based vectors for insertion of the gfp marker gene or for delivery of cloned DNA into Gram-negative bacteria at a neutral chromosomal site. J Microbiol Methods. 2001;45(3):187-195.

4. Choi KH, Gaynor JB, White KG, Lopez C, Bosio CM, Karkhoff-Schweizer RR, et al. A Tn7-based broad-range bacterial cloning and expression system. Nat Methods. 2005;2(6):443-448.

5. Miyazaki R, Minoia M, Pradervand N, Sulser S, Reinhard F, van der Meer JR. Cellular variability of RpoS expression underlies subpopulation activation of an integrative and conjugative element. PLoS Genet. 2012;8(7):e1002818.
